# Simplification and Shift in Cognition of Political Difference: Applying the Geometric Modeling to the Analysis of Semantic Similarity Judgment

**DOI:** 10.1371/journal.pone.0020693

**Published:** 2011-06-06

**Authors:** Junko Kato, Kensuke Okada

**Affiliations:** 1 Graduate Schools for Law and Politics, The University of Tokyo, Bunkyo, Tokyo, Japan; 2 Department of Psychology, School of Human Science, Senshu University, Kawasaki, Kanagawa, Japan; University of Zaragoza, Spain

## Abstract

Perceiving differences by means of spatial analogies is intrinsic to human cognition. Multi-dimensional scaling (MDS) analysis based on Minkowski geometry has been used primarily on data on sensory similarity judgments, leaving judgments on abstractive differences unanalyzed. Indeed, analysts have failed to find appropriate experimental or real-life data in this regard. Our MDS analysis used survey data on political scientists' judgments of the similarities and differences between political positions expressed in terms of distance. Both distance smoothing and majorization techniques were applied to a three-way dataset of similarity judgments provided by at least seven experts on at least five parties' positions on at least seven policies (i.e., originally yielding 245 dimensions) to substantially reduce the risk of local minima. The analysis found two dimensions, which were sufficient for mapping differences, and fit the city-block dimensions better than the Euclidean metric in all datasets obtained from 13 countries. Most city-block dimensions were highly correlated with the simplified criterion (i.e., the left–right ideology) for differences that are actually used in real politics. The isometry of the city-block and dominance metrics in two-dimensional space carries further implications. More specifically, individuals may pay attention to two dimensions (if represented in the city-block metric) or focus on a single dimension (if represented in the dominance metric) when judging differences between the same objects. Switching between metrics may be expected to occur during cognitive processing as frequently as the apparent discontinuities and shifts in human attention that may underlie changing judgments in real situations occur. Consequently, the result has extended strong support for the validity of the geometric models to represent an important social cognition, i.e., the one of political differences, which is deeply rooted in human nature.

## Introduction

The expression of differences in terms of spatial analogies appears to be intrinsic to human cognition. Geometric models constitute one of the representational–computational views of mind but have maintained a low profile [1] compared to other mental representation models (i.e., symbolism and associationism, particularly connectionism). Based on Minkowski geometric modeling, multidimensional scaling (MDS) has primarily analyzed the similarity judgment data related to visual and auditory sensations [2]–[5]. The judgment of abstract differences in semantics lies close to the core of human intelligence but is hard to analyze with geometric modeling. Modeling the analysis of semantic differences requires assignment of a real number, known as a *distance*, to represent the (dis)similarity between the objects in terms of meanings that are more subtle than sensations. Reasoning and/or taxonomy when obtaining semantic similarity judgment data tend to depend exclusively on experimental controls that differ among studies [6]–[9] and may not have immediate relevance to real social contexts.

We solved both the theoretical and empirical problems described above by analyzing judgments on differences associated with political entities. In everyday situations, people often express political (dis)similarity in spatial terms. The first recorded instance of this linguistic practice dates to the French revolution in 1789 [10]. On this historic occasion, the emergence of order in the national assembly, which was characterized by a variety of beliefs and opinions, apparently went hand in hand with spatial positioning. “There is a Right Side (*Coté Droit*), a Left Side (*Coté Gauche*); sitting on M. le President's right hand, or on his left; the *Coté Droit* conservative; the *Coté Gauche* destructive” ([10], p. 192]). Here, spatial and political terms found simultaneous expression in the right-sided seat assignments of “conservative” party members and the left-sided seat assignments of members of the “destructive (progressive)” parties.

A considerable literature has reported that people in politics, regardless of time and space, tend to locate themselves and political parties on a scale with extreme positions on either the right or left end ([11], p. 209) and to express differences between positions as “distances”. Building on universal observations, political economists have developed spatial models for use in analyses of real practices in which political positions are represented as points in space [12], [13] and have attempted to quantify different positions with respect to a left–right ideology and substantive policies in terms of distance [14]–[19]. Survey questionnaires, in which respondents are asked to rate political positions on scales, have provided data that can be regarded as equivalent to experimental data on similarity judgments [20]. Political economists, however, have never regarded these data as ideal for the analysis of human cognition, whereas cognitive scientists have yet to find semantic similarity judgment data that are immediately relevant to practices in society. We used political survey data for the MDS analysis based on Minkowski geometric models.

### A parallel between geometric and psychological modeling

Distinct metrics have been used in the geometric modeling of cognitive space (i.e., mapping similarity judgments of perceptual symbols such as colors, sounds, numbers, and shapes). Euclidean, city-block, and dominance metrics are members of the general Minkowski family of distance metrics, and city-block and dominance metrics represent two of the most popular non-Euclidean metrics [21]. In this context, MDS, which is a statistical technique for data analysis, may well be regarded as a framework for modeling human cognition [22]. The Minkowski distance of order *p* (*p*-norm distance) between objects *i* and *j* judged by the *k-th* subject (individual) is defined by:
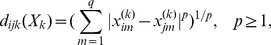
(1)where 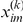 and 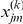 are the 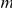-th dimension's coordinates of object points 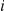 and 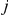, respectively, for subject *k*. When 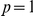, Equation (1) defines city-block distances; when 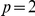, it defines Euclidean distances; and when *p* = *∞*, it defines dominance distances. More specifically, the relationship of Minkowski distances to dimensions is referred to in terms of a “dimensionality” that represents the degree of the influence of the dimensions on the definition of distance. Dimensionality decreases as the value of *p* surpasses 1 and approaches 2 (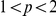) and is totally lost when 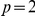. This property is familiarly known as the Pythagorean Theorem. Dimensionality starts to increase when the value surpasses 2 (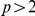) and is restored when *p* approaches infinity.

**Figure 1:**
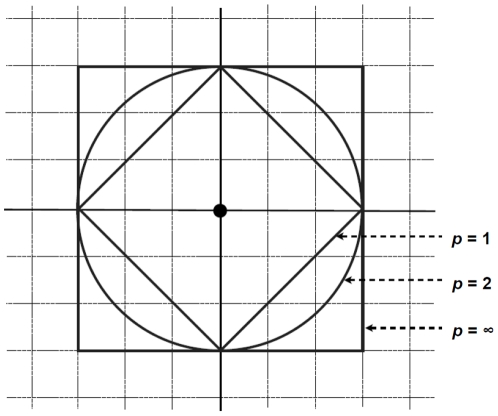
Isosimilarity contour of the origin for different *p*-values in the Minkowski distance formula (**Equation 1**).

Figure 1 shows an isosimilarity contour, a set of points that are equidistant from the origin, of three metrics in two-dimensional (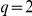) space. The diamond-shaped contour of the city-block metric resulted from a set of equidistant points in the squared city-block (or grid). According to the Euclidean metric, the Pythagorean Theorem proves that this is a circle and that the distances are invariant if orthogonally rotated. As *p* increases to infinity, a maximum component distance (i.e., a maximum of 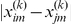 among 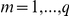 dimensions) solely determines the distance, 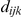. According to the dominance metric, the dimension with the highest summand “dominates” the definition of distance ([23], pp. 22–23). More intuitively, differences between two points ( = component distances) are suppressed in all dimensions except in the one that maximally discriminates among them. In two-dimensional space, the isosimilarity contour of the city-block metric is congruent with that of the dominance metric after a 45-degree rotation stretching a factor of the square root of 2, as shown in Figure 1. As cited by Arabie ([21], p. 574), this is a special case of the isometry between a city-block metric of *q* dimensions and a dominance metric of 2*^q-1^* dimensions that Koopman and Cooper (Text S1) proved more generally and has attracted special attention in cognitive psychology ([24], p. 357; [25], pp. 406–7). This isometric relationship involves an important implication for interpreting the present results, which will be introduced later.

The geometric property of a distinct metric can be regarded as corresponding to the particular cognitive space that serves to map stimuli. The Euclidean metric predicts the cognitive spaces (and/or distances) of *unitary* stimuli with *integral* dimensions better, and the city-block metric predicts the cognitive space of *analyzable* stimuli with *separable* dimensions better [26]. According to scales completed by subjects, the pitch of a sound is always consistent with a particular loudness and the dimensions representing pitch and loudness levels are integrated. In tasks placing objects on a similarity scale, a value for one dimension (e.g., saturation) cannot be judged in the absence of a value for another (e.g., hue or brightness). In such cases, dimensions are regarded as integral to perceptions. Individuals are not conscious of *integral* dimensions, which are elaborated to analyze data obtained with similarity scales for *unitary* stimuli; thus, these dimensions exist only in experiments and analyses. In contrast, *analyzable* stimuli with *separable* dimensions emerge in daily perceptual experiences. If color chips differ in both size and saturation, for example, they are analyzable, subject to scaling along separable dimensions representing size and saturation, respectively (cf. [1], p. 24). When graphic symbols (e.g., triangles) differ in size and orientation, they are also classified as analyzable stimuli with differences scaled along separable dimensions.

Garner ([4], p. 120) distinguished integral dimensions involved in a “primary” process of perception from a separable dimension involved in a “secondary” perceptual process that is more derivative or cognitive. More concretely, Garner argued that people do not immediately distinguish the hue, saturation, and brightness of colors and, thus, their scaling consists of a set of integral dimensions even though analysis, as exemplified by the Munsell color system, can eventually differentiate the respective values of the integral dimensions. This differentiation between primary and secondary processes produces a continuum rather than a dichotomy involving ostensibly distinct psychological spaces. In originally proposing the utility of the Minkowski metric, Torgerson ([27], pp. 292–293) noted the possibility that “in many situations, the subject's judgment falls between,” the Euclidean and city-block metrics. Shepard ([26], p. 55) concurs with Torgerson on this point.

### Semantic similarity judgments

Non-semantic similarity judgments relating to the five senses are verified by external and quantifiable measures independent of human cognition, irrespective of the distinctions discussed above. The perceived intensity/strength of sensory stimuli is not necessarily proportional to the physical magnitude of the stimuli and, thus, the reported perceptions may diverge from those reflecting physical quantities (i.e., the Weber–Fechner law). The subjective intensity/strength of stimuli can be compared with the physical magnitude of stimuli quantified by external measures such as weight, hertz, decibels, time, and so on (i.e., Stevens' formula). However, it is impossible to find the corresponding external measures to quantify semantic stimuli and, thus, we have no way of comparing the subjective values of these stimuli using measures that are external to human perception. In this regard, the analysis of semantic stimuli is clearly distinguished from that of non-semantic stimuli. The semantic dimensions in the cognitive space of similarity judgments are “*phenomenal*, aimed at describing the psychological structure of the perceptions” but not “*scientific*, where the structure of the dimensions used is often taken from some scientific theory” ([1], p. 5). Individuals are expected to measure semantic “distances” based on intellectual interpretations of events, texts, and objects and, thus, judgments relating to semantics are more amenable to change and revision, depending on context as well as on accompanying conditions.

As illuminated above, the dimensions of the semantic differences are not defined *a priori* to human perception, whereas the dimensional structures of differences in sensory stimuli (e.g., colors and tones) are confirmed by relating them to quantifiable measures that are external to the human cognition ([28], p. 328). In this context, semantic similarity judgment hinges critically on finding the low-dimensional structure that cannot be verified independently of the cognitive process. Consequently, the MDS generation of the semantic dimensions emerges as equivalent to the cognitive process of identifying a relevant low-dimensional space that would have been otherwise buried in the high-dimensional observations of semantic differences. The MDS analysis of political data enables us to explore the critical process underpinning semantic similarity judgments.

## Materials and Methods

### Materials

We analyzed the data obtained from the “expert survey on party positions” that are available to the public at http://www.politics.tcd.ie/ppmd/. This survey was conducted between 2002 and 2004 in 51 democratic countries and asked political scientists to judge the (dis)similarity of the political positions of parties in each country. The survey method, thoroughly explained elsewhere [19], ensured that the data were collected in a way that used experimental controls comparable to those available in a cognitive psychology laboratory.

- Procedure: Questionnaires were mailed or available on the web to enable voluntary participation. The survey was conducted after the general elections in each country.
- Subjects: The sample of respondents was chosen from a directory or list, provided by a national political science association, of the political scientists in each country.
- Object 1: Party Position on Policies: All respondents were asked about the parties' positions on all policies considered by the survey organizers to constitute critical issues in each country's political domain. Politicized policies were not entirely the same across countries. As a result, certain policy issues were included for the survey in all or most countries, whereas others were specific to one or several countries.
- Object 2: Party Ideological Position: All respondents were also asked to judge each party's position on the left–right ideological scale in each country.
- Scales for judging positions: Scales ranging from 1 to 20, varying by increments of 1, were used to rate each party's position. Scaled distances between party positions were regarded as representations of their differences. The most leftist position is designated as 1 on the left end and most rightist position is designated as 20 on the right end of ideological scale. On each policy scale, the extreme position that has been usually (and/or in most countries treated) as “left” is designated as 1 on the left end and the opposite extreme position that has been treated as “right” is designated as 20 on the right end.

Table 1 (modified based on Table A1 in [19]) presents the details of the survey design for the 13 countries used in our analysis. For this analysis, we included 13 countries that (1) had at least five parties, (2) had at least seven policies to examine, and (3) had at least seven experts who responded to questions about the positions of all parties with regard to all policies (with no missing data); thus, we started the MDS analysis with more than 245 dimensions in each country's dataset. Table 2 presents an example of the data obtained from the first three respondents in Germany, who rated the policy positions and ideologically scaled position (the rightmost column) of each of 10 parties with regard to seven distinct policies. The survey provided distinct three-way datasets pertaining to 1) *experts, political parties, and policies* and 2) *experts, political parties, and ideology*, obtained from survey results from 13 countries.

**Table 1:** Survey design for the 13 countries used in our analysis.

	Questionnaire				Respondents		
			Total	Total	Total	Total	Response
Country	Language	Format	Parties	Dimensions	Respondents	Surveyed	Rate (%)
Canada	English	Web	6	10	104	611	17%
Denmark	English	Web	10	9	26	54	48%
Germany	German	Web	10	9	98	525	19%
Hungary	Hungarian	Web	8	13	42	124	34%
Israel	English	Web	12	8	30	185	16%
Italy	Italian	Web	13	10	54	182	30%
New Zealand	English	Web	8	8	21	73	29%
Norway	English	Web	8	9	21	37	57%
Portugal	Portuguese	Web	6	9	21	73	29%
Spain	Spanish	Web	5	10	76	381	20%
Sweden	English	Web	7	10	67	244	27%
Switzerland	French/German	Paper	10	8	51	197	26%
United Kingdom	English	Web	5	11	57	145	39%

**Table 2:** An example of the data obtained from the first three respondents in Germany.

		Decentralization		EU	EU	EU			Taxes V	Ideological
Expert	Party		Environment	Accountability	Authority	Peacekeeping	Immigration	Social	Spending	Scaling
1	DKP	15	10	6	17	16	10	8	3	4
1	PDS	10	3	6	10	16	10	8	3	3
1	GR	3	6	3	11	5	3	2	14	6
1	SPD	7	11	12	11	8	9	8	7	9
1	FDP	3	14	5	9	5	3	2	17	11
1	CDU	7	15	13	9	7	9	16	11	12
1	Sch	10	13	15	15	12	16	15	13	15
1	Rep	14	13	16	17	17	18	17	10	18
1	DVU	14	13	16	17	17	18	10	10	19
1	NPD	14	13	16	17	17	18	10	10	18
2	DKP	5	8	1	12	6	8	4	2	3
2	PDS	7	10	1	14	8	4	1	1	5
2	GR	5	4	1	8	6	7	5	11	8
2	SPD	8	9	9	11	8	10	7	10	10
2	FDP	8	14	9	10	6	11	6	20	13
2	CDU	8	15	11	13	8	18	18	19	14
2	Sch	6	19	5	20	20	20	18	8	18
2	Rep	6	18	5	20	20	20	19	9	19
2	DVU	6	17	5	20	20	20	20	9	19
2	NPD	6	19	5	20	20	20	20	8	20
3	DKP	8	3	2	8	19	4	2	3	1
3	PDS	8	3	2	6	18	2	2	3	3
3	GR	6	5	6	9	6	6	3	10	8
3	SPD	14	9	7	9	5	7	10	7	6
3	FDP	9	17	8	10	5	8	6	19	16
3	CDU	13	15	13	10	5	16	15	13	14
3	Sch	4	11	14	12	4	20	20	16	20
3	Rep	4	11	14	12	4	20	20	16	20
3	DVU	4	11	14	12	4	20	20	16	19
3	NPD	4	11	14	12	4	20	20	16	20

Several methodological problems may have influenced the results. The first relates to the distinction between two sets of three-way data involving ideology and policy, respectively. Political analysts have repeatedly confirmed that position on the left–right ideological scale, as opposed to position on specific policies, constitutes a general criterion for judging overall general political position [29]–[34]. At the same time, however, the existing literature reports that the left–right ideological scaling provides a rough approximation for judging overall policy positions, but does not entirely represent each policy position. More specifically, the level of (statistically significant) correlation between the left–right scaling of parties and the scaling of party policy positions varied greatly between policies and across and within countries [19], [20], [30]. Building on this empirical finding, we used the three-way dataset involving multiple policy positions in the MDS analysis to identify the cognitive space for political difference and the three-way dataset involving ideologies to explore the meaning of the MDS dimensions generated by the analysis of the policy data.

Second, the survey selectively chose respondents from a specific group of political scientists who had become accustomed to translating “differences” between positions into “distances” for the purpose of political analysis. Ordinary people adopt spatial analogies in daily conversation but may not be sufficiently conscious of analogous “differences” to express these in terms of scaled “distances”. Although geometric cognition may be used by both experts and non-experts, higher and lower levels of geometric cognition may characterize experts and non-experts, respectively. In this regard, selecting political scientists as respondents may have inadvertently underscored unconscious spatial cognition.

Third, in contrast to other psychological experiments, this study addressed differences in real entities that are not under full experimental control. Survey responses might not represent similarity judgments on only the positions addressed in the survey. Because political parties have had immediate political relevance to respondents, respondents' sympathies for specific parties, which may be expected to increase as these positions move closer to their own, may have systematically biased responses. To examine this issue, the survey included a question using the same scale ranging from 1 (most sympathetic) to 20 (least sympathetic) about the extent of “sympathy” for all parties. Benoit and Laver ([19] , pp. 90–92) confirmed the absence of statistically significant correlations between ratings on positions and sympathy at the individual level.

### Method

Our data can be summarized as three-way dissimilarity data, in which one dissimilarity matrix between parties was obtained from each expert in the data set for each country. Our analysis aimed at clarifying which Minkowski metric, the Euclidean or the city-block, fits better with the cognitive space in which the similarity judgments were reached. The standard statistical methodology for analyzing the metric structure is nonmetric MDS, which we also performed for the data for each country. MDS had been used intensively in the psychological experiments on similarity judgments conducted from the 1970s to the 1990s [21]. However, methodological researchers recently found two major problems with the Minkowski metric MDS: (1) the problem of information loss resulting from taking the average dissimilarity, and (2) the problem of local minima in the optimization of the loss (stress) function. In what follows, we explain each problem and discuss how we overcame the associated difficulties.

Regarding the first problem, a common strategy of MDS analyses has been to average across subjects to obtain a single summarized dissimilarity matrix (in our case, a dissimilarity matrix per country; see, e.g., [35]). However, previous studies found that averaging these data exerted a misleading basis on modeling [36]–[38]. Thus, a good fit of an MDS model “to averaged data cannot be taken as evidence that the model describes the psychological structure that characterizes individual subjects ([37], p.144).”

To avoid the difficulty of averaging across subjects, we decided to apply a three-way MDS technique to our three-way data. Specifically, we used a *weakly constrained* MDS [22], [39], which can be viewed as one of the three-way weighted Euclidean models. The major difference between the standard MDS and the weakly constrained MDS lies in the loss function (stress) to be optimized. In a standard MDS model, the configuration matrix is computed to minimize the badness-of-fit measure known as stress or, more specifically, as Kruskal's stress-1, which is defined as:
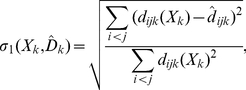
(2)where 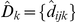 is the target disparity matrix of 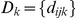. On the other hand, the badness-of-fit measure in a weakly constrained MDS is defined as
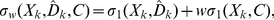
(3)where 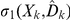 is the loss of configuration 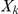 relative to constraint matrix 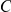, which is identical within each country, and 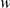 is a nonnegative weight, which we set at 100 according to the suggestion of Borg and Groenen [22]. The second term in Equation (3) is a penalty term, which penalizes configurations that do not satisfy the constraint. For the constraint matrix 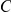, we used the average country dissimilarity matrix, which works as a weak constraint that renders the configuration within a country more uniform. Note that our constraint was a *weak* one, in the sense that it did not strictly restrict the solution, but just penalized those solutions that did not satisfy the constraint.

The second difficulty with MDS involves local minima in the optimization of the loss function. Recent methodological studies have found serious problems of local minima and degeneracy in the computation of ex-standard steepest descent MDS algorithms, especially when using the city-block metric (e.g., [40]–[42]). Because of the local minima problem, some results of previous studies that tried to calculate stress for non-Euclidean metrics might be incorrect. For example, Groenen et al. [41] analyzed the cola data of Green, Carmone, and Smith [43] and found that existing algorithms fell into local minima, especially when *p* was close to 1. Additionally, Okada, Kato, and Shigemasu [44] found that the well-known results reported by Kruskal [35], who analyzed Ekman's [45] color data, failed to minimize stress, especially when the Minkowski metric was 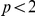, which included the city-block metric.

Our analysis was not immune from the problem of local minima. To ensure finding the global minimum, we adopted several recently developed methodologies to optimize the stress function to avoid local minima. First, we used a *distance smoothing* technique, which was proposed by Pliner [46], [47] and extended to any Minkowski metric by Groenen et al. [42]. By smoothing the spiky peak of the distance function, this technique helps the optimization algorithm avoid many local minima. In fact, the numerical example shown in Groenen et al. [42] suggested the superior performance of smoothing over that of conventional methods used in previous studies. Second, we used a *majorization* algorithm [48], in which the optimization problem reduces to the optimization of a so-called majorizing function. This method is known to be better than the common steepest descent algorithm in terms of guarantees for, and rates of, convergence in optimization. The majorization algorithm was extended to Minkowski distances by Groenen et al. [42], [49] Third, we established 50 different random starting values for each analysis to further avoid the effect of local minima.

In sum, for the three-way data of a country, the loss function of weakly constrained MDS (Equation 3) was minimized by the majorization algorithm with distance smoothing. This was repeated 50 times per country with different random starting values, and the calculation that resulted in the lowest stress was used as a final result. Smooth 4.0 [42] software as well as the *R* statistical environment [50] were used for conducting the aforementioned analyses. No *post-hoc* rotation of the configuration was performed.

To check the meaning of each dimension, we calculated Kendall's correlation coefficients (Kendall's *tau*) between the coordinates of city-block dimensions and the ideological scaling scores for each subject. We did this only for the city-block solution because the indeterminacy of rotation remained in the Euclidean solution. In the MDS with Euclidean distance, the rotation of the dimension does not change the value of stress but does change the correlation between one of the dimensions and some other external criterion. However, this problem is inherent in Euclidean distance and does not occur at other Minkowski distances, including the city-block distance. Kendall's *tau* coefficient was chosen as a measure of relationships because our analysis, as well as most applications of MDS in the social sciences, utilized nonmetric MDS. In nonmetric MDS, only the rank order of each variable in the data is assumed to contain the essential information. Nonmetric MDS is often used in applications in psychology and social science because the data in these areas are often considered to include nonnegligible noise. Because Kendall's *tau* is also a nonparametric measure of association based on rank order, it is considered to be more appropriate than parametric measures, such as Pearson's correlation coefficient.

## Results

The results can be summarized by the following three observations.

### Reducing the number of dimensions to two

**Figure 2:**
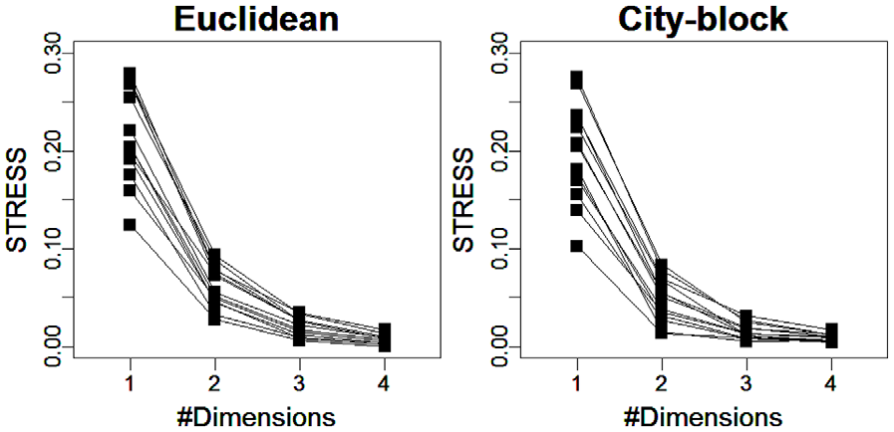
Stress value versus number of dimensions for 13 countries (one line corresponds to one country). (left) Euclid metric (right) City-block metric.

The number of dimensions was reduced to two when fit was calculated in terms of stress. We fit the three-way MDS model described above to each individual dataset and then calculated the average stress for each country. Figure 2 shows the plot of stress values versus the number of dimensions, which was manipulated from one to four, in the city-block and Euclidean metrics. Each line corresponds to one of the 13 countries. In most countries, the value of stress dropped dramatically from one to two dimensions and then gently descended from two to four. Additionally, the stress values were smaller than 0.1 for all countries. A common criterion for interpreting stress is that stress below 0.1 indicates a good fit (e.g., [51], [52]]. Given that this criterion was met for all countries, we can say that the MDS yielded a good fit in an only two-dimensional solution for our data.

### The better fit of the city-block metric than the Euclidean one

**Figure 3:**
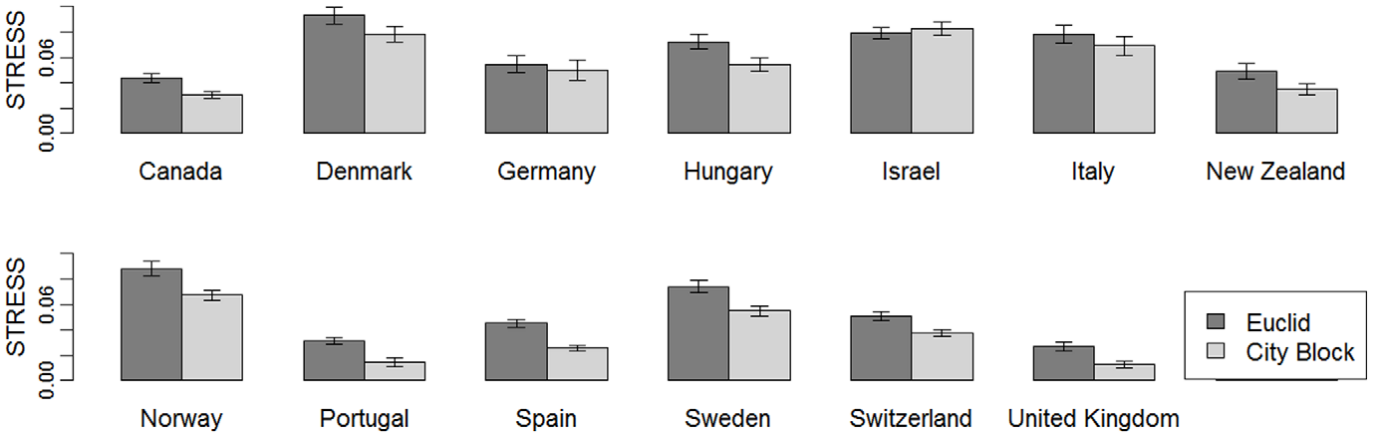
Mean (±SE) stress for each country in Euclidean and city-block metrics.

We next compared the stress value of the Euclidean and city-block metrics in a two-dimensional MDS analysis. Figure 3 shows the mean (±standard error) stress value for each country. The error bars correspond to standard errors. For all countries but Israel, the city-block metric provided a lower mean stress value than did the Euclidean metric.

### Correlation with the ideological scaling

**Figure 4:**
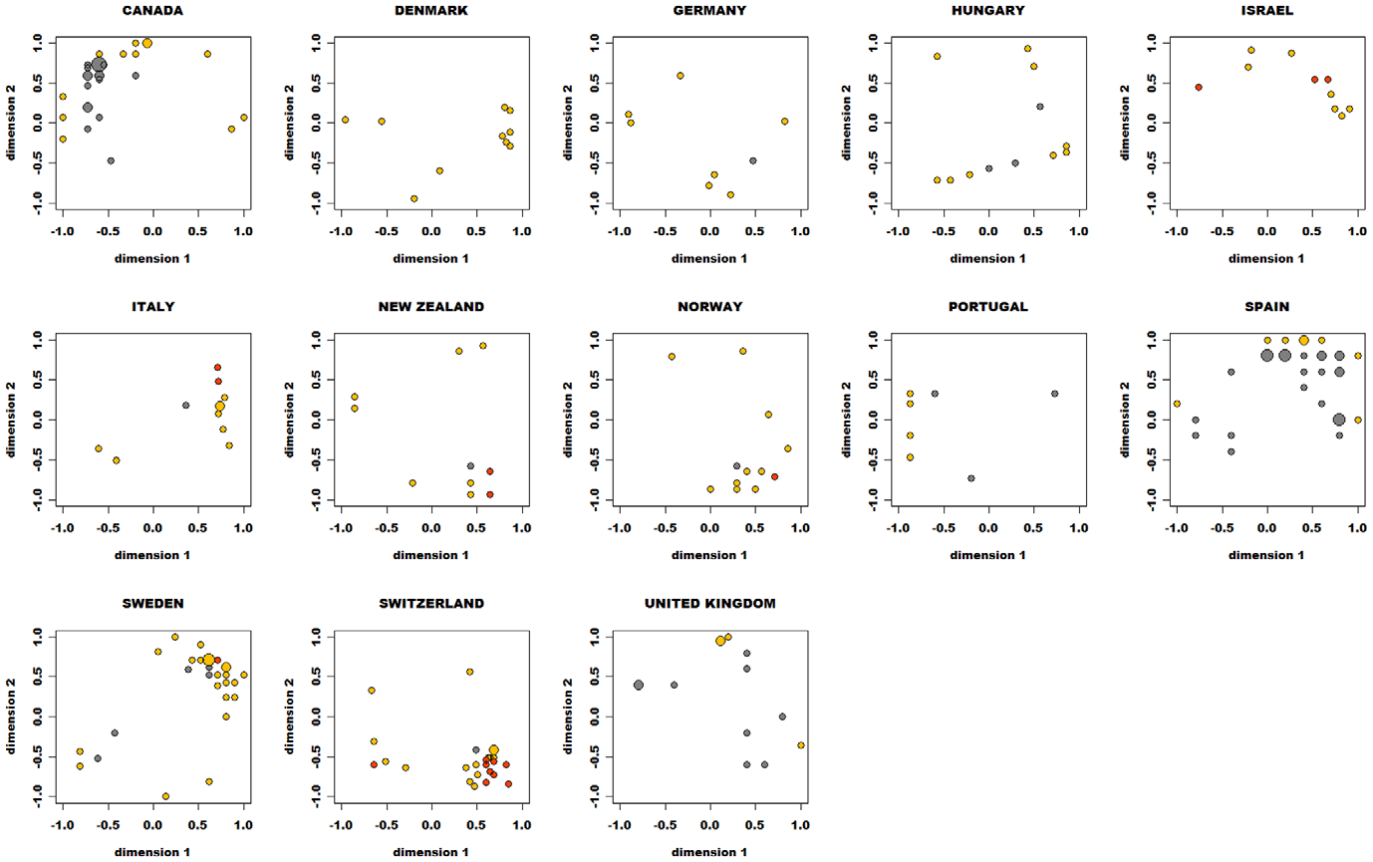
Scatterplots of the Kendall's correlation coefficient of each expert between the MDS dimensions and the left–right ideology scale in the survey. Orange color indicates statistical significance (p<.05) of one (of two) dimension, and red color indicates statistical significance (p<.05) of both dimensions.

At least one city-block dimension in most of the configurations tended to have a statistically significant correlation coefficient with a high absolute value with the left–right ideological scaling in the survey. Figure 4 shows the scatterplots of resultant Kendall's correlation coefficients with the color indicating the statistical significance (p<.05). In nine countries (the exceptions being the United Kingdom, Canada, Switzerland, and Spain), the correlations of at least one of the two dimensions were statistically significant in most configurations. In the cases of the United Kingdom, Canada, and Spain, the correlation of neither dimension was statistically significant in more than two-thirds of the configurations. On the other hand, in Switzerland, the coefficients of both dimensions were significant in 60% of the configurations and tended to be either positively or negatively correlated with the left–right dimension measured in the survey.

## Discussion

The analysis provides two implications immediately drawn from the results and one inferred from the geometric property of the Minkowski metric. First, the reduced number of dimensions implies that a variety of differences between objects may be summarized and simplified when judging their (dis)similarities. Second, according to the city-block solution that was superior to the Euclidean approach for mapping perceived differences, most of the generated MDS dimensions may be closely related to the criterion (i.e., the left–right ideology) that we actually use in real political situations to simplify differences. Lastly, the inference drawn from the geometric property of the Minkowski metric penetrates into a critical component of the cognitive process. The generated city-block metric has an isometric relationship with the dominance metric in a two-dimensional space (Text S1). The isometry may geometrically represent a shift in attention and point to a discontinuity in the cognition of political differences.

### The cognitive relevance of the two-dimensional city-block metric

In the absence of a priori specifications of valid qualitative dimensions, the analysis ensured a drastic reduction in the number of dimensions that constituted the cognitive space for similarity judgments. Specific policy differences that were originally represented by more than 245 dimensions may involve parallels. We inferred that respondents had been integrating policy differences, which are geometrically represented in the same way, into identical dimensions. Such convergent cognitive processes may plausibly operate when people judge real political differences in society.

The city-block metric, which yielded the better fit with cognitive space than did the Euclidean, has recently attracted the special attention of theorists working on the spatial modeling of politics. The spatial modeling that has been dominated by the Euclidean metric predicts the absence of an equilibrium point chosen by a simple majority rule (i.e., a point that is closer than any other point to as many points as possible) in two or more dimensional space [53]. Imposing strong assumptions (on the distribution of points representing preferences as well as voting/aggregating rules) enables one to theoretically identify an equilibrium [54], [55]. Despite this theoretical practice, however, we have not yet observed as much difficulty in real decision-making. Spatial modeling may be appropriately applied to the non-Euclidean metric. Extending the previous findings [56], [57] in this domain, Humphreys and Laver [58] recently proved the presence of a majority-rule equilibrium with coordinates that represent medians in all dimensions (i.e., the dimension-by-dimension median) of high-dimensional city-block metrics. As Humphreys and Laver explicate, however, the validity of the aforementioned prediction depends on whether real humans actually evaluate political similarities and differences using city-block distances. Our results have provided further evidence supporting the empirical relevance of the city-block metric [59]–[61].

### The empirical relevance of city-block dimensions to real judgments

The MDS dimensions, which presumably indicate differentiated ways for judging (dis)similarity, per se, have no predicated meanings and thus may be difficult to interpret. Another three-way dataset pertaining to ideology, however, suggests a straightforward interpretation of results. One of the city-block dimensions in most of the generated MDS configurations tends to be highly correlated with the left–right scaling obtained from the survey and, thus, may plausibly represent the criterion (i.e., ideology).

Left–right positioning, which has been conventionally and universally used to simplify political differences, has posed a puzzle for political analysts. Despite its prevalence, it is hard to find a priori parallels in enduring political divisions manifesting across a variety of cases that correspond to left and right positioning [11], [19]. The substantive meaning of left–right ideologies is highly context-dependent and the referents of the left–right ideological scale may change according to time and circumstances. Our results may suggest that the use of the left–right distinction may result from the same sort of cognitive simplification of differences by which all semantic differences tend to be linearly contrasted and geometrically represented for the purpose of making judgments.

### Representing cognitive shift in the isometric city-block and dominance metrics

Dimensionality remains important in the city-block metric. The dominance metric reduced the definition of distance to one dimension. Cognitive psychologists have speculated that the isometry of their sharp-cornered isosimilarity contours imply a shift and a discontinuity in human similarity judgment and, thus, in cognition ([21], pp. 569–71, 574). More specifically, their isometric relationship implies a shift from a two-dimensional cognitive space (i.e., represented in the city-block metric) to a one-dimensional one (i.e., represented in the dominance metric) and vice versa. The geometric property, at first glance, appears too abstract to understand human cognition, let alone have major consequence for human behavior. However, this isometric relationship may represent a discontinuity in cognition and provide a consistent explanation for a shift from judgments that divide attention between distinct issues to judgments focused on one issue, and vice versa ([21], pp.569–70). For example, a person's judgment and choice often change broadly in social matters, including politics, and the discontinuity may accompany a shift in attention. Despite the importance of the puzzle, the absence of empirical evidence has precluded exploring the geometric property of non-Euclidean metrics ([21], pp. 569–571, 577). Our analysis, by identifying the two-dimensional city-block MDS configuration, underscores the possibility that the observed shift in human cognition may be a result of the form of the metric used to represent cognitive space. Geometric modeling of cognitive space may explain the underlying cognitive process associated with a frequently observed shift characterizing popular attention in real politics that has been considered as lacking apparent continuity or rules.

Spatial thinkers in political science and economics have used the Euclidean metric without seriously examining the relevance of non-Euclidean geometric models for real human judgments. Cognitive scientists have been unable to obtain the relevant data for exploring semantic similarity judgments and have decreased the attention devoted to Minkowski (i.e., non-Euclidean) metrics. The empirical evidence presented in this study can help fill the lacunae that impede our understanding of the cognitive processes relevant to real social contexts. Our result has extended strong support for the validity of the geometric models to represent an important social cognition, i.e., the one of political differences, which is deeply rooted in human nature.

## Supporting Information

Text S1 **Koopman RF, Cooper M (1974)** ***Some Problems with Minkowski Distance Models in Multidimensional Scaling.*** Paper presented at the meeting of the Psychometric Society, Stanford, CA. Uploaded with permissions from both Koopman RF and Cooper M.
(PDF)
