# Supplementary material for: Simplification and Shift in Cognition of Political Difference: Applying the Geometric Modeling to the Analysis of Semantic Similarity Judgment
Source: PLoS One. 2011 Jun 6;6(6):e20693. doi: 10.1371/journal.pone.0020693 (PMC3108968; doi:10.1371/journal.pone.0020693)

Paper presented to the Psychometric Society, 28 March 1974, Stanford, Ca.

Some problems with Minkowski distance models in multidimensional scaling

Raymond F. Koopman and Mary Cooper
Simon Fraser University

We start by considering the end points of the Minkowski continuum, city-block space ($r = 1$) and dominance space ($r = \infty$), and assert that any set of interpoint distances in a city-block space of m dimensions can be reproduced exactly by a configuration of points in a dominance space of $m^* = 2^{m-1}$ dimensions.

If A is an n x m matrix giving the coordinates of n points in a city-block space of m dimensions, then the n x m* matrix A* giving the coordinates of the points in a dominance space of m* dimensions may be obtained by taking $A^* = AH$, where H is a selection of m rows from the general Hadamard matrix of order m*. The general expression for the elements of H is

$$h_{pq} = (-1)^{(q-1)/(2^{m-p})} ,$$

where the division operation in the exponent should be taken as yielding an integer quotient, dropping any remainder. H is composed entirely of +1's and -1's. The p'th row of H consists of 2^{p-1} runs of +1's and -1's, with each run of length 2^{m-p}. Each row starts with a +1. A sample H for m = 4 is given below.

$$\begin{matrix} 1 & 1 & 1 & 1 & 1 & 1 & 1 & 1 \\ 1 & 1 & 1 & 1 & -1 & -1 & -1 & -1 \\ 1 & 1 & -1 & -1 & 1 & 1 & -1 & -1 \\ 1 & -1 & 1 & -1 & 1 & -1 & 1 & -1 \end{matrix}$$

Each column represents one of the 2^{m-1} possible permutations of sign of m-1 unit elements in rows 2 through m; all such permutations are present, and none are duplicated.

The proof of our assertion now follows quite easily. The distance between points i and j in city-block space is given by

$$d_{ij} = \sum_p |a_{ip} - a_{jp}|$$
$$= \sum_p |c_{ijp}| .$$

The distance between points i and j in the dominance space obtained from city-block space by the above transformation is given by

$$d^*_{ij} = \max_q |a^*_{iq} - a^*_{jq}|$$
$$= \max_q \left| \sum_p a_{ip} h_{pq} - \sum_p a_{jp} h_{pq} \right|$$
$$= \max_q \left| \sum_p (a_{ip} - a_{jp}) h_{pq} \right| = \max_q \left| \sum_p c_{ijp} h_{pq} \right|$$
$$= \max_q |c^*_{ijq}| .$$

Since the columns of H represent exactly half of the 2^m possible permutations of sign of m elements, there will always be at least one column, say q', in H in which the signs are either all the same as the signs of the c_{ijp} or all opposite to the signs of the c_{ijp}. (If $c_{ijp} \neq 0$ for all p, then there will be exactly one such column q'.) Clearly then $|c^*_{ijq'}| = d_{ij}$. Moreover, since $|c^*_{ijq'}| \geq |c^*_{ijq}|$ for all $q \neq q'$, we have $|c^*_{ijq'}| = d^*_{ij}$, and therefore $d^*_{ij} = d_{ij}$, which was what we set out to prove.

Thus, any 2-dimensional city-block space can be transformed into a 2-dimensional dominance space without altering any of the interpoint distances. Similarly, 3- and 4-dimensional city-block spaces can be transformed into 4- and 8-dimensional dominance spaces, respectively.

This relation is not generally reversible. That is, even if a dominance space is of a dimensionality which can be expressed in the form 2^{m-1} for some integer m, it will not generally be possible to transform it into a city-block space without also altering the interpoint distances: although the rows of H are orthogonal, the columns are linearly dependent unless m = 2. This allows an alternative interpretation of A* as the projections of the points onto m* non-orthogonal axes placed through the city-block space by the transformation H. When looked at this way, it is seen that each dominance axis is a vector normal to a pair of opposing faces of the city-block isodistance surface.

In 2 dimensions, however, H is square and both row- and column-orthogonal. In this case, it represents a rigid 45° rotation and uniform contraction of the axes, and an inverse transformation does exist. Here we have a perfect example of the paramorphic representation problem: in 2 dimensions it is impossible to tell whether a city-block rule or a dominance rule is appropriate, since each is a transformation of the other mathematically, in spite of the fact that they may be quite different psychologically. In higher dimensionalities the problem still exists, but not to the same degree: in order to replace a city-block space by a dominance space, we have to be willing to put up with an increase in the number of dimensions.

So far we have considered only the limiting values of the metric constant. Do similar results hold for intermediate values $1 < r < \infty$? In general, we do not know. We can say, though, that if any 2-dimensional solution with a metric constant r is rotated 45° and given an appropriate amount of expansion or contraction, and if a new metric constant $r^* = r/(r-1)$ is used, then the ratio of the distance d^* between any two points in the transformed space over the corresponding distance d between the same two points in the original space will satisfy the inequality

$$\frac{1}{1.01635} \leq \frac{1}{c_t} \leq \frac{d^*}{d} \leq c_t \leq 1.01635 ,$$

where c_t is a constant that depends on r. We do not have a strict mathematical proof of this statement; the demonstration depends on numerical, rather than analytic, results.

It will be noted that the formula given above for r^* implies that

$$1/r + 1/r^* = 1 .$$

Therefore r and r^* always lie on opposite sides of 2. It is also possible to express both r and r^* as functions of a single parameter, say t:

$$r = 1 + e^{t}$$
$$r^* = 1 + e^{-t} .$$

Since $t = 0$ generates $r = r^* = 2$, which are Euclidean spaces, and since non-zero values of t generate conjugate metric constants which approach 1 and infinity as t approaches ±infinity, it may be useful to consider the magnitude of t as an index of "non-Euclideanness". The constant c_t referred to above is related to the absolute value of t, and is tabled on the next page.

t	r	r^{a}	c_t	
0	2	2	1	
.25	2.28	1.78	1.0043	
.50	2.65	1.61	1.0083	
.75	3.12	1.47	1.0116	
1.00	3.72	1.37	1.0141	
1.25	4.49	1.29	1.0157	
1.50	5.48	1.22	1.0163	
1.58	5.85	1.21	1.01635	— maximum
1.75	6.75	1.17	1.0162	
2.00	8.39	1.14	1.0155	
2.50	13.2	1.08	1.0129	
3.00	21.1	1.05	1.0098	
3.50	34.1	1.03	1.0070	
4.00	55.6	1.02	1.0048	

There are no local extrema other than the one indicated. Since $c_t \to 1$ as $t \to \infty$, our previous result showing the equivalence of 2-dimensional city-block and dominance spaces is seen to be a special case of the present situation.

Ordinarily, however, the "closeness" of two configurations is not measured by a relative error index such as we have given above, but by a normalized sum of squared absolute errors. To see what kind of alternate solution might be obtained when such a function is minimized, we constructed a set of distances from a 10-point ellipse using a metric constant of 1.8, and looked at the sum of squared absolute errors, minimized with respect to the coordinates of the points and normalized by the sum of squares of the input distances, at a number of different values of r. The true solution and the best of the alternate solutions are given in Figure 1, and a plot of the error as a function of r is given in Figure 2. The best alternate solution was found at r = 2.24, which is not too far from the value of 2.25 which we would predict, and the closeness of the alternate solution to a 45° rotation of the true solution is immediately obvious. One somewhat surprising aspect of this data is that the fit is very good regardless of the value of r. In terms of goodness of fit alone, any one of the solutions would do quite well.

It is interesting to speculate whether one or more such locally-optimum alternate solutions may exist in higher-dimensional problems. We have no theoretical reasons for saying so, only a strong hunch that they probably do. Out of curiosity, we took a 3-dimensional configuration, calculated interpoint distances using a metric constant of 1.6, and went looking for alternate solutions at different values of r. The original coordinates, and those of the best alternate solution with an $r \geqslant 2$, are given in Table 1. A plot of the error as a function of r is given in Figure 3. The cusp at $r = 1.45$ appears to be real; no amount of fiddling with the minimization algorithm (Fletcher-Powell) could make it disappear. In any case, there is definitely a local minimum on the side of 2 opposite to the true solution.

In general, and most certainly for 2 dimensions, our results suggest that the interval $1 \leq r \leq 2$ may be mapped into the interval $2 \leq r \leq \infty$. This is a rather surprising result if the traditional interpretation of the psychological meaning of the metric constant is accepted. This interpretation is that when $r = 1$, the component differences between two objects are simply added to determine their total dissimilarity. As r increases from 1 toward infinity, more and more weight is given to the larger component differences, until at $r = \infty$, only the largest difference is taken into account in determining the total dissimilarity. This interpretation implies a continuity over the interval $(1, \infty)$ which is not consistent with our findings.

A recent alternative interpretation suggested by Micko & Fischer is slightly more consistent. Without going into their theory, it can be said that it does involve a sort of interchangeability between values of r which are on opposite sides of 2, in that certain weightings which they postulate will be identical. The side of 2 on which r lies is postulated to be a function solely of whether an additive or a dominance rule of combination is being used; no other rules apply. However, our results sugggest that even this distinction is arbitrary, each rule beigg a transformation of the other.

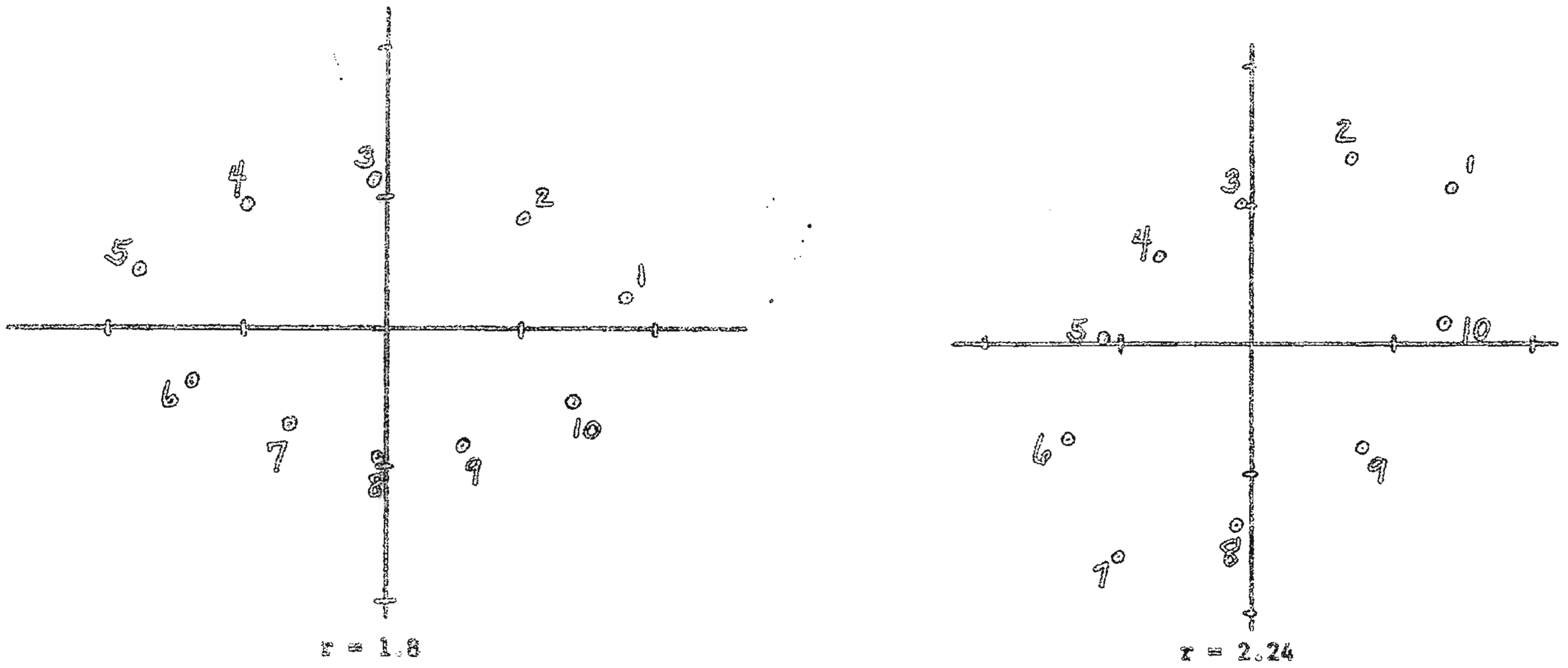

Figure 1

The true ($r = 1.8$) and alternate ($r = 2.24$) solutions for errorless data

Figure 2

Error as a function of r for the ellipse data

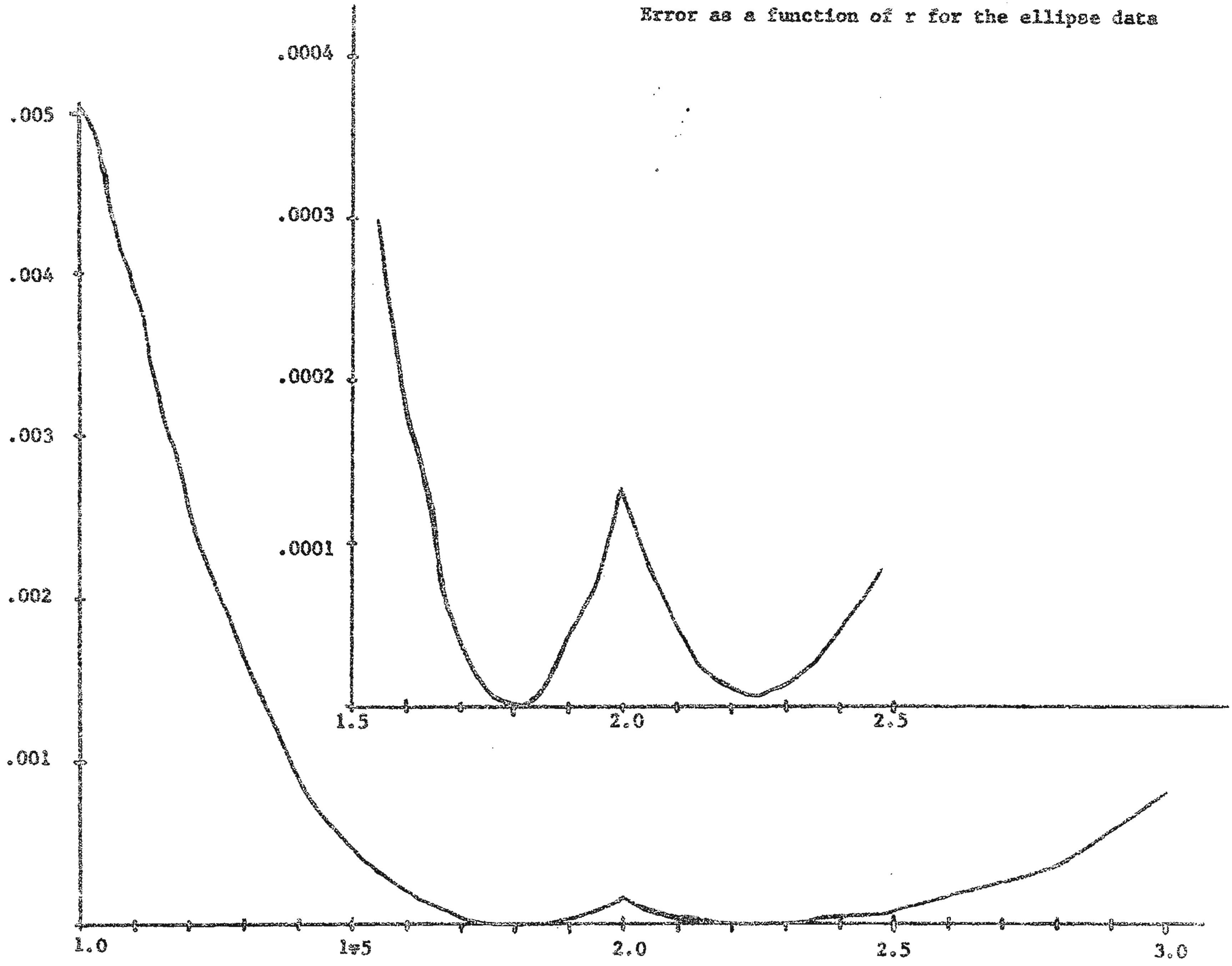

Table 1

The true (r = 1.6) and alternate (r = 2.26) solutions for errorless data

r = 1.6

.7168	.1110	-.4203
.8293	-1.2106	-.1961
-.7225	.6012	.1042
.1814	.1049	-1.4724
.2432	-.4339	-.2366
-1.4086	-.9760	.8831
-.8755	.7407	.3148
1.0359	1.0627	1.0232

r = 2.26

.8613	-.2127	.3280
.0993	-.9494	1.3676
-.3089	.4812	-.9617
1.1259	-1.1165	-.6299
.0811	-.5737	.3407
-2.1867	-.1514	-.0698
-.4903	.7931	-1.0556
.8184	1.7290	.6808

Figure 3

Error as a function of r for 3-dimensional data

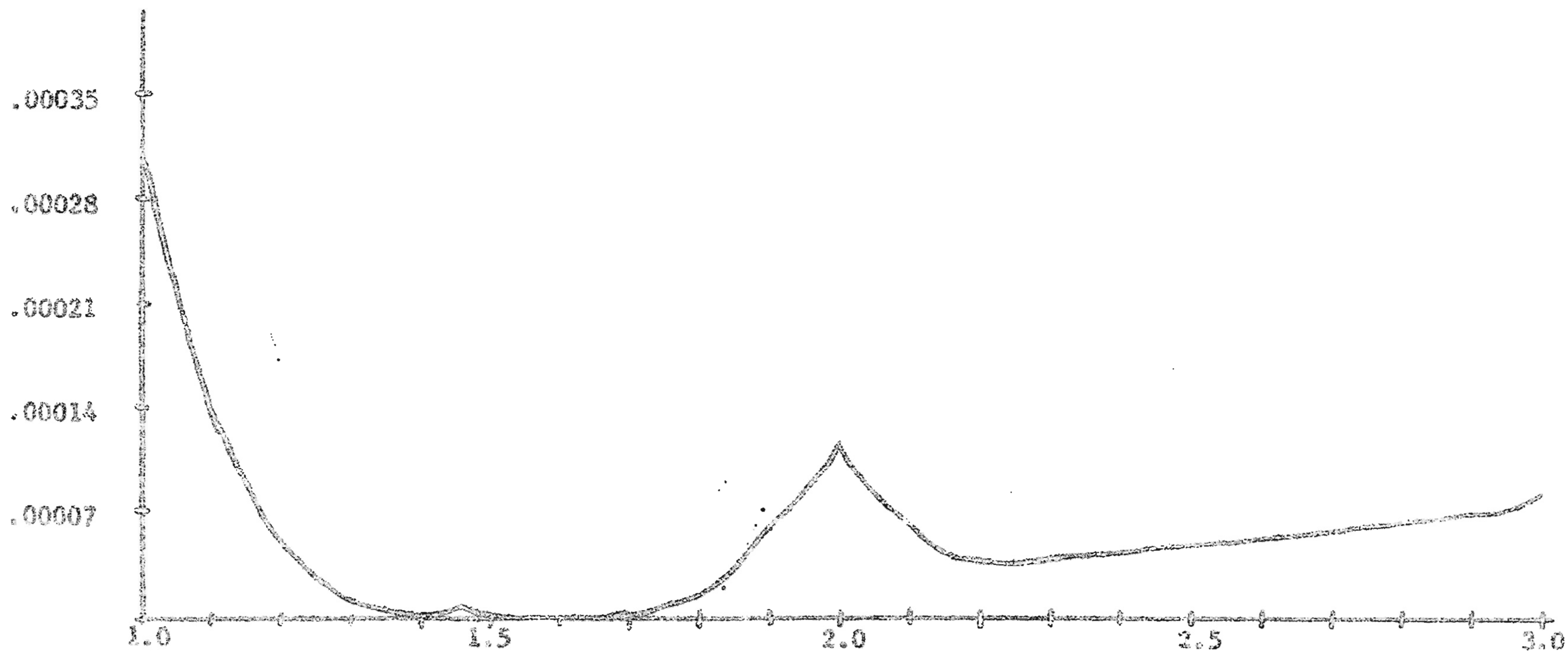

Supplement: Text S1 — Koopman RF, Cooper M (1974) Some Problems with Minkowski Distance Models in Multidimensional Scaling. Paper presented at the meeting of the Psychometric Society, Stanford, CA. Uploaded with permissions from both Koopman RF and Cooper M. (PDF) [file pone.0020693.s001.pdf]
